# Pathology

**Published:** 1846-03

**Authors:** 


*Pathology.—Cuiious Alteration of the Cornea in Hydrocephalus Acutus.*—Dr. Stöber, of Strasburg, has observed, in several cases of hydrocephalus acutus, shortly before death, a peculiar alteration of the cornea of the eye. It consists in a semilunar yellow speck, situated at the lower margin of the cornea, without undue redness or vascularity of the eye itself. This morbid alteration gradually extended upwards, when the disease was protracted, to the centre of the cornea, and terminated in ulceration. The lamellæ of the cornea were separated by a purulent infiltration, and pushed outwards, and in this way were detached from each other by the ulceration. Dr. Stöber regards this process as the consequence of the general debility. In one case, where the prostration of strength was considerable, he directed the instillation of opium drops (opiumo draáber) into the eye, and prescribed some doses of quinine, with a nourishing diet. These means were successful, and in ten days the ulceration had cicatrised.—*Ibid.*

